# Anesthesia for Suboccipital Craniotomy in a Patient with Lymphangioleiomyomatosis: A Case Report

**DOI:** 10.1155/2012/804789

**Published:** 2012-07-29

**Authors:** Robert A. Peterfreund, Emily Luman, Robert L. Martuza

**Affiliations:** ^1^Department of Anesthesia, Critical Care and Pain Medicine, Massachusetts General Hospital, Harvard Medical School, Boston, MA 02114, USA; ^2^Department of Neurosurgery, Massachusetts General Hospital, Harvard Medical School, Boston, MA 02114, USA

## Abstract

Lymphangioleiomyomatosis (LAM) is a rare pulmonary condition often presenting with spontaneous pneumothorax. Imaging or biopsy confirm the diagnosis. Published case reports describe the anesthetic management of patients with LAM undergoing brief procedures. No reports describe the anesthetic management for lengthy neurosurgical procedures. We describe anesthetic management for craniotomy in a patient with LAM. *Clinical Features*. A woman presented with 2 spontaneous left pneumothoraces. She received a diagnosis of LAM by imaging. She did well after pleurodesis. Hearing loss and tinnitus led to brain imaging demonstrating a large left cerebello-pontine angle mass. She presented for elective craniotomy to remove the mass while preserving cranial nerve function. Our technique for general endotracheal anesthesia aimed to reduce the likelihood of another pneumothorax while providing good surgical conditions and permitting neuromonitoring. *Conclusion*. We demonstrate the successful anesthetic management of a patient with LAM undergoing a lengthy suboccipital craniotomy for a posterior fossa mass.

## 1. Introduction

Lymphangioleiomyomatosis (LAM) is a rare pulmonary condition manifested by cyst formation in the lungs. Previous reports describe anesthesia management for patients with LAM needing brief surgical procedures, typically in the abdomen or chest. We present the anesthesia management of a patient with LAM undergoing a lengthy suboccipital craniotomy.

LAM primarily affects the lung. Proliferation of smooth muscle-like cells produces obstruction of the vasculature, the lymphatics, and the airway resulting in pulmonary cyst formation [1, 2]. Other organs, particularly the kidney, may also be affected. The prevalence of LAM is about 1 : 1,000,000 in the general population, predominantly in women. The initial presentation is typically with respiratory symptoms, often with pneumothorax as a physical finding. The precise etiology of LAM remains undetermined, but an association with tuberous sclerosis suggests a common genetic cause [3–5].

A patient with a large suboccipital mass presented for craniotomy. We were confronted with the question of how to anesthetize this patient. Only a few case reports found in a PubMed search discuss management of patients with LAM requiring general anesthesia. The majority of these reported patients had relatively brief obstetric, abdominal, or thoracic procedures [6–13]. The literature search returned no publications describing a patient undergoing a lengthy and complex neurosurgical procedure. With the patient's written informed consent, we now report the anesthetic management of a patient with a diagnosis of LAM presenting for suboccipital craniotomy and resection of a large cerebello-pontine angle (CPA) mass lesion.

## 2. Case Description

**Figure 1:**
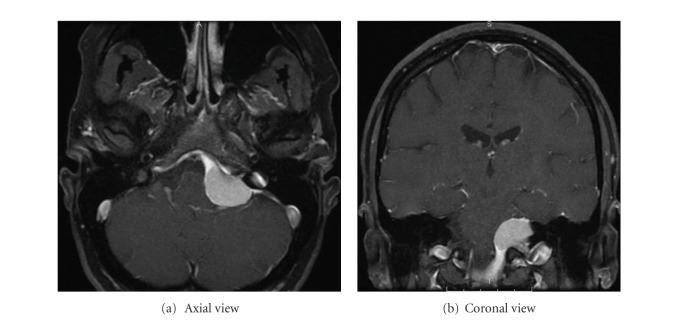
Preoperative axial and coronal MRI images of the patient's brain. Gadolinium enhancement demonstrates a large mass in the left cerebello-pontine angle.

A middle-aged woman initially presented to an outside hospital with a spontaneous left pneumothorax. A second left pneumotharax was treated by ipsilateral pleurodesis. The diagnosis of LAM was made by imaging, without a specific tissue diagnosis. She had a good respiratory status and managed to do limited exercise. Recent pulmonary function tests were essentially normal. Room air oxygen saturation was 99%. She had a history of breast cancer in remission but was otherwise healthy and took no medications. One year prior to surgery, she noted left side tinnitus and partial hearing loss without other neurological symptoms. Brain imaging identified a left CPA mass extending caudally into the jugular foramen (Figure 1). She was referred to our institution for evaluation and possible treatment. A pulmonary physician consulted at our institution concluded that the patient could undergo anesthesia and surgery with careful attention to respiratory parameters. The resulting surgical plan was for a retromastoid approach to resect the mass, with a major goal of preserving nerve function, including cranial nerve V, VII, VIII, and X.

The major focus of the anesthesia plan was to avoid rupturing a pulmonary cyst which might create another pneumothorax, especially on the right (untreated) side. We also needed to provide appropriate conditions for exposure and dissection of the mass while the surgeons monitored cranial nerve function by direct stimulation. This required us to conduct most of the anesthesia without the use of muscle relaxants. After insertion of a peripheral IV catheter, the patient received midazolam, 2 mg, IV. Routine monitors were applied; these were supplemented by a radial artery line placed after anesthesia induction. After routine IV induction of general anesthesia (propofol, fentanyl), a mask airway was easily established with careful attention to limit inflation pressures to less than 20 cm of water. Succinylcholine facilitated routine oral intubation with a NIM^TM^ endotracheal tube (Medtronic, Minneapolis, MN, USA) by direct laryngoscopy. This endotracheal tube was chosen to allow motor monitoring of cranial nerve X via contractile activity of the laryngeal musculature. The maintenance anesthetic consisted of isoflurane (Fi 1.2–1.8%) in a mixture of air and oxygen, supplemented during the early part of the case with intermittent bolus doses of fentanyl. Towards the end of the operation, we began an infusion of remifentanil (0.05–0.1 mcg/kg/min). To allow motor monitoring of cranial nerves V, VII, and X, the patient did not receive any additional relaxants after intubation. Hearing (CN VIII) was also monitored. Mannitol and furosemide provided adequate brain relaxation for surgery.

**Figure 2:**
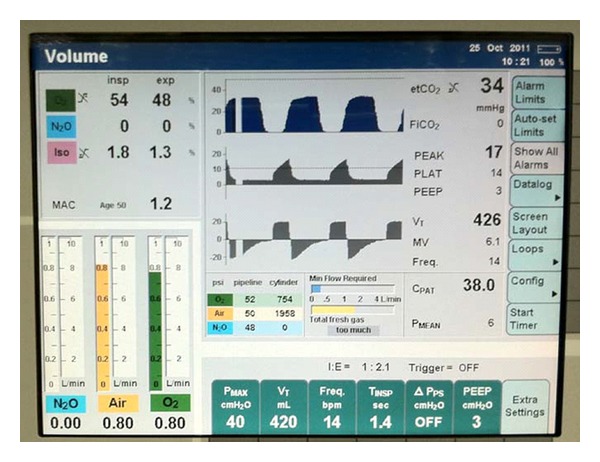
Screen shot of intraoperative ventilator parameters and measurements. The image depicts fresh gas flows, expired and inspired concentrations of isoflurane, airway pressures and flows, and expired CO_2_ tracings. Tidal volume was set at approximately 6 mL/kg. The anesthesia machine was an Apollo from Dräeger (Pittsburgh, PA).

The ventilator was set for volume control ventilation at 6 mls/kg tidal volume, 10–14 breaths/minute, with PEEP (3 cm of water). These settings maintained oxygenation and ventilation with peak airway pressures less than 20 cm of water. Serial arterial blood gas determinations (FiO_2_ 52–58%) demonstrated good oxygenation with the PaO_2_ ranging between 179 and 195 at or near normal pH. EtCO_2_ was controlled in the range of 33–37 without capnographic evidence of airway obstruction (Figure 2).

Biopsy of the mass revealed a meningioma. To preserve cranial nerve function, surgery was limited to a subtotal resection. Towards the end of the ~9-hour surgical procedure, the patient received hydromorphone (1.4 mg) in divided doses for postoperative analgesia along with antiemetics (haloperidol and ondansetron). The patient was emerged from anesthesia and uneventfully extubated in the operating room. Neurological examination demonstrated preserved cranial nerve function, including hearing. Two postoperative chest radiographs demonstrated no pneumothoraces. The patient had no respiratory complaints. Room air oxygen saturations on the first postoperative day ranged from 92 to 98%. The patient developed a CSF leak treated with a lumbar drain. Otherwise, she did well and went home 9 days after surgery. With the exception of some nausea, she had no concerns related to anesthesia.

## 3. Discussion

To our knowledge, this is the first paper describing anesthesia management of a patient with LAM undergoing craniotomy. Key goals of the anesthetic were to maintain oxygenation with ventilation appropriate for craniotomy, to facilitate monitoring of the cranial nerves at risk for injury, and to provide a quiet surgical field for delicate microsurgery without the use of muscle relaxants. Some of these considerations differ from the anesthesia management concerns for other types of procedures, as described in previous reports [7, 8, 12, 13].

To prevent pneumothorax, inflation pressures were carefully kept at a low level. Although nitrous oxide is commonly used at our institution for elective craniotomy procedures when intracranial pressure is normal, we chose to avoid this agent to reduce the likelihood of rupturing a closed lung cyst. Anesthetic depth sufficient to maintain a quiet surgical field, hemodynamic stability, and good gas exchange with a fast and smooth emergence was readily accomplished with isoflurane, fentanyl, and a remifentanil infusion.

We, thus, conclude that at least some patients with LAM can safely undergo lengthy major neurosurgical procedures with careful attention to respiratory management.
